# Does Whole-Blood Neutrophil Gelatinase-Associated Lipocalin Stratify Acute Kidney Injury in Critically Ill Patients?

**DOI:** 10.1155/2019/8480925

**Published:** 2019-05-02

**Authors:** M. Cuartero, A. J. Betbesé, K. Núñez, J. Baldirà, J. Ordonez-Llanos

**Affiliations:** ^1^Intensive Care Department and Institut d'Investigacions Biomèdiques, Hospital de la Santa Creu i Sant Pau, Universitat Autònoma de Barcelona, Barcelona, Spain; ^2^Biochemistry Department and Institut d'Investigacions Biomèdiques, Hospital de la Santa Creu i Sant Pau, Universitat Autònoma de Barcelona, Barcelona, Spain

## Abstract

**Purpose:**

To analyse the capacity of whole-blood NGAL (wbNGAL) to stratify AKI in critically ill patients with and without sepsis.

**Methods:**

Whole-blood NGAL was measured with a point-of-care device at admission and 48 hours later in patients admitted to a general ICU. Patients were classified by the AKIN and KDIGO classifications at admission and 24 and 48 hours. We performed an ROC curve analysis. wbNGAL values at admission were compared in patients with sepsis and septic shock.

**Results:**

The study included 100 consecutively admitted patients (40 female) with mean age 59.1 ± 17.8 years. Thirty-three patients presented AKI at admission, and 10 more developed it in the next 48 h. Eighteen patients had AKI stage 3, 14 of them at admission. Nine patients required renal replacement therapy. According to KDIGO at admission, wbNGAL values were 78 *μ*g/L (60-187) in stage 0 (*n* = 67), 263 *μ*g/L (89-314) in stage 1 (*n* = 8), 484 *μ*g/L (333-708) in stage 2 (*n* = 11), and 623 *μ*g/L (231-911) in stage 3 (*n* = 14), *p* = 0.0001 for trend. Ten patients did not complete 48 hours of study: 6 of 10 were discharged (initial wbNGAL 130 *μ*g/L (60-514)) and 4 died (773 *μ*g/L (311-1010)). The AUROC curve of wbNGAL to predict AKI was 0.838 (95% confidence interval 0.76-0.92, *p* = 0.0001), with optimal cut-off value of 178 *μ*g/L (sensitivity 76.7%, specificity 78.9%, *p* < 0.0001). At admission, twenty-nine patients had sepsis, of whom 20 were in septic shock. wbNGAL concentrations were 81 *μ*g/L (60-187) in patients without sepsis, 481 (247-687) in those with sepsis, and 623.5 *μ*g/L (361-798) in the subgroup of septic shock (*p* < 0.0001).

**Conclusions:**

Whole-blood NGAL concentration at ICU admission was a good stratifier of AKI in critically ill patients. However, wbNGAL concentrations were higher in septic patients irrespective of AKI occurrence.

## 1. Introduction

Acute kidney injury (AKI) is a disease often diagnosed in intensive care unit (ICU) patients. AKI incidence can be as high as 24% depending on its definition [1]. ICU-related AKI is associated with an in-hospital mortality that can reach 60% [2, 3]. Therefore, early AKI detection is crucial to prevent or stop the natural course of renal dysfunction and improve its morbi-mortality. Serum creatinine is the gold standard biomarker in all AKI definitions [4–6]. However, creatinine is a late biomarker for AKI, as values peak after 24-48 hours of renal injury. Hence, several biomarkers have been proposed for early AKI detection [7, 8].

Neutrophil gelatinase-associated lipocalin (NGAL) is a glycoprotein first isolated from specific granules of human leukocytes [9]. NGAL was found elevated in bacterial infections when compared to viral infections [10]. NGAL exists as a monomeric form of 25 kDa, a homodimer linked by a disulphide bridge of 45 kDa and a heterodimer with matrix metalloproteinase-9 (MMP-9, gelatinase) with an intermolecular disulphide bridge of 135 kDa [11]. NGAL is also formed in other cells apart from leukocytes. In response to several injuries, NGAL is expressed in kidney, hepatic, or epithelial cells [11–13]. In the kidneys, NGAL is freely filtered by the glomerulus and reabsorbed in the proximal tubule [14]. After a tubular injury, NGAL is overexpressed in the diseased endothelium [15–17]. In this setting, tubular reabsorption is reduced, and blood and urine NGAL concentrations increase. In AKI, NGAL increase is observed 24-48 hours earlier than plasma creatinine peak [18, 19]. However, the specificity of NGAL to detect AKI could be limited by overexpression in other tissues [20]. This could be relevant in critically ill patients, in whom sepsis could promote NGAL release from tissues other than renal epithelium [21, 22]. NGAL has been described as a predictor of AKI and the need of renal replacement therapies (RRT) in patients admitted to general intensive care units (ICU) [21–24]. However, as a systematic review by Hjortrup et al. [24] pointed out, the NGAL role is not fully understood: literature shows that NGAL might predict AKI with a wide range AUROC, from 0.54 to 0.98. The aims of this study were (1) to evaluate the capacity of whole-blood NGAL at ICU admission to predict AKI development and (2) to analyse the effect of sepsis on its predictive capacity.

## 2. Methods

### 2.1. Patients

The study protocol was approved by the Ethic Committee Board at Hospital de la Santa Creu i Sant Pau (Barcelona, Spain). We obtained informed consent from participants or their guardians. The study prospectively included 100 patients consecutively admitted during 8 months (June 2010-February 2011) in a general ICU. Inclusion criteria were age over 18 years old and the expected ICU stay of at least 48 hours. Patients were excluded if they had already been admitted in the hospital for more than 24 hours, had any degree of preexisting chronic kidney disease, or were not expected to survive for at least 24 hours due to a nonreversible clinical condition. Physicians attending patients were blinded to NGAL results throughout the study.

### 2.2. Clinical and Laboratory Data Collection

Clinical data included admission diagnosis, demographics, ICU severity scores, haemodynamic parameters, and urine output. Patients were initially classified following the AKIN definition [5] and KDIGO [6] definition for AKI. Both definitions were based on serum creatinine and urine output obtained since admission to 6 hours later, at 24 and 48 hours of admission. Baseline serum creatinine was taken from patients' preadmission records whenever possible and used to estimate eGFR before ICU admission using the Cockcroft–Gault formula. According to standard criteria [25], patients were also classified as having sepsis and septic shock.

NGAL was measured at admission and 24 and 48 hours in EDTA-anticoagulated whole-blood using the Triage® NGAL Test (Alere Diagnostics, formerly Inverness Medical Innovations). All samples were analysed in the same batch to avoid between-batch variability.

### 2.3. Statistical Analysis

SPSS® version 18 (SPSS Inc., Chicago, IL) was used. Variables with normal distribution are reported as mean ± standard deviation and were compared with Student's *t*-test or one-way analysis of variance. Variables with non-Gaussian distribution are reported as median and interquartile range (IQR) and were compared with the Mann–Whitney *U* or Kruskal-Wallis tests. Categorical data are reported as percentage and were compared by the chi-square test or Fisher exact test. Reporting of results followed the STARD (Standards for Reporting Diagnostic Accuracy Studies) statement. Whole-blood NGAL predictive values were evaluated by receiver operating characteristic (ROC) curve analysis. We defined an area under the ROC (AUROC) curve of 0.60-0.69 as poor, 0.70-0.79 as fair, 0.80-0.89 as good, and 0.90-1.00 as excellent in terms of predictive value. A *p* < 0.05 was considered significant.

## 3. Results

### 3.1. Clinical Characteristics

**Figure 1:**
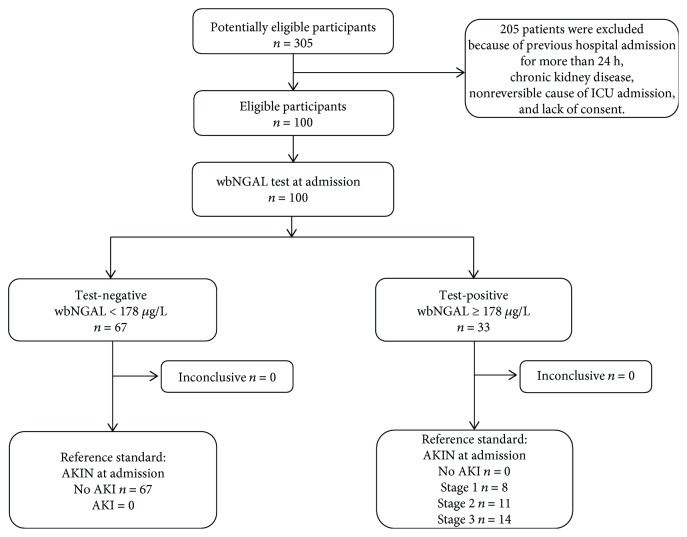
Diagram of whole-blood NGAL to predict AKI, study enrolment and inclusion/exclusion criteria.

We recruited 100 consecutive patients fulfilling the admission criteria (Figure 1). Ten of them did not complete the 48 h follow-up due to discharge (6 cases) or death (4 cases). Mean age was 59.1 ± 17.8 years. 60% of cases were male. The causes of ICU admission were medical 54% (respiratory 26%, cardiovascular 7%), postsurgical care 39% (gastrointestinal 21%, neurosurgery 12%), and miscellaneous 7%. At ICU admission, twenty-nine were septic, and twenty of them had septic shock. ICU length of stay was 10.3 ± 9.6 days, and ICU mortality was 22%.

### 3.2. Whole-Blood NGAL and AKI

**Figure 2:**
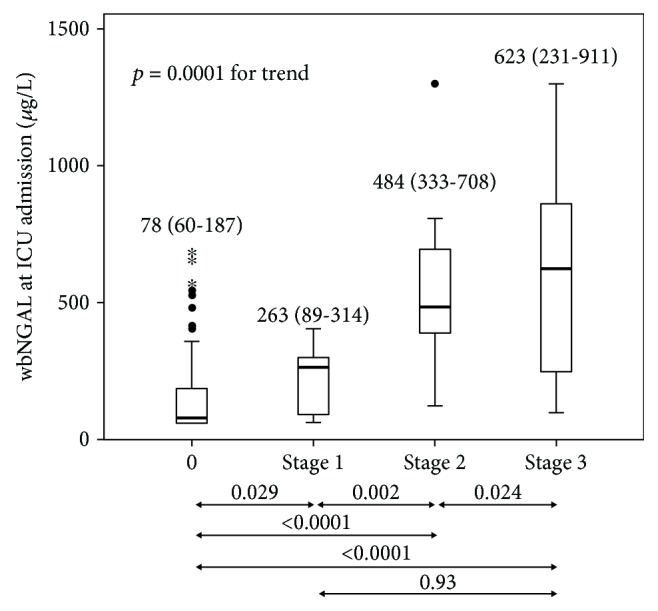
Boxplot comparing whole-blood NGAL (wbNGAL) concentrations (*μ*g/L) and KDIGO score at admission. Boxplots indicate the median and 25^th^ and 75^th^ percentiles. Whiskers indicate the 5^th^ and 95^th^ percentiles. Statistical significance (*p*) comparing wbNGAL (*μ*g/L) with KDIGO categories is given at the bottom of the figure.

Forty-three patients presented AKI, 33 at admission (8 stage 1, 11 stage 2, and 14 stage 3) and 10 more within 48 h of the ICU stay; 4 of the latest group of 10 developed renal failure (stage 3). Nine patients required renal replacement therapies. wbNGAL values were 78 *μ*g/L (IQR 60-187 *μ*g/L) in non-AKI patients and 263 *μ*g/L (IQR 89-314 *μ*g/L), 484 *μ*g/L (IQR 333-708 *μ*g/L), and 623 *μ*g/L (IQR 231-911 *μ*g/L) in those with stage 1, stage 2, and stage 3, respectively (*p* = 0.0001 for trend) (Figure 2). Four of 33 patients with AKI at admission were diagnosed of AKI solely because of urine output criterion (one patient had stage 1, one patient had stage 2, and two patients had stage 3); 10 of 33 patients were diagnosed of AKI based on changes in sCr, and the remaining 19 patients were diagnosed based in both sCr and drop of urine output. The incidence and severity of AKI were the same when AKIN classification was applied within the first 48 h.

In the group of 6 patients discharged before 24 h, admission wbNGAL and plasma creatinine were of 130 *μ*g/L (IQR 60-514) and 78 *μ*mol/L (IQR 54-123), respectively; whereas in the 4 patients who died, wbNGAL was of 773 *μ*g/L (IQR 311-1010) and plasma creatinine 165 *μ*mol/L (IQR 59-577). We did not find statistical differences in wbNGAL or serum creatinine between subgroups of patients who were discharged or passed away.

**Figure 3:**
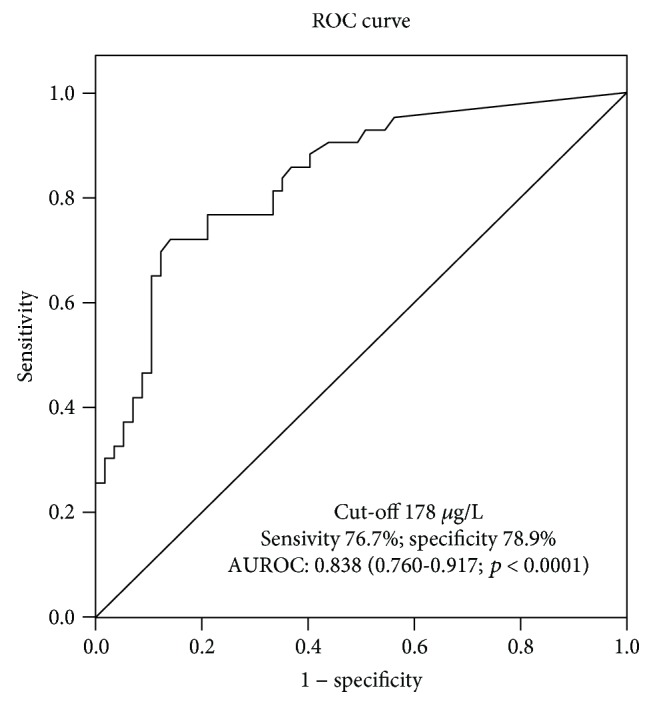
ROC curve for whole-blood NGAL value at ICU admission to predict AKI. Diagnostic and overall accuracies given with sensitivity, specificity, and 95% confidence interval compared with gold standard KDIGO classification.

Whole-blood NGAL values were predictive of AKI both at admission and within 48 h of the ICU stay (Supplemental Table 1). The area under the ROC curve (AUROC) of wbNGAL for AKI prediction within 48 hours of ICU admission was 0.838 (95% confidence interval (CI) 0.760-0.917, *p* = 0.0001). The wbNGAL optimal cut-off for AKI within 48 h of ICU admission was 178 *μ*g/L, with sensitivity of 76.7% and specificity of 78.9% (Figure 3). The AUROC of admission serum creatinine for AKI within the first 48 h of ICU admission was 0.904 (95% CI 0.841-0.967, *p* = 0.0001). There were no statistical differences between wbNGAL and plasma creatinine AUROC.

Forty-five patients had wbNGAL > 178 *μ*g/L. They were more likely to be older, have higher SOFA at admission, have higher incidence of AKI development, stage 3, sepsis, or septic shock, and have requirement of vasopressor drugs and renal replacement therapies during the ICU stay (Table 1). Six of eighteen patients without AKI at admission but wbNGAL > 178 *μ*g/L developed AKI within the next 48 h. Accordingly, wbNGAL identified at admission extra 14.6% of AKI patients not diagnosed by the serum creatinine criterion.

### 3.3. Whole-Blood NGAL and Sepsis

**Figure 4:**
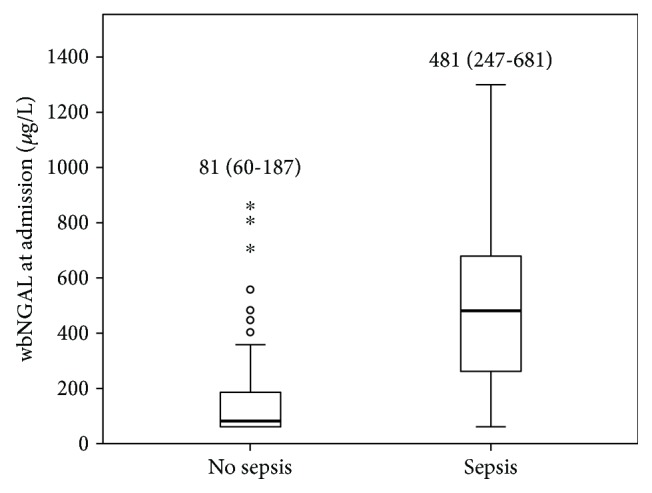
Whole-blood NGAL concentrations (*μ*g/L) according to the presence of sepsis. Boxplots indicate the median and 25^th^ and 75^th^ percentiles of wbNGAL in ng/L. Whiskers indicate the 5^th^ and 95^th^ percentiles. Statistical significance (*p*). Patients without sepsis *n* = 71; patients with sepsis *n* = 29.

wbNGAL concentrations were 81 *μ*g/L (IQR 60-187) and 481 *μ*g/L (IQR 247-681) in 71 patients without sepsis and 29 patients with sepsis, respectively (*p* < 0.0001) (Figure 4). wbNGAL was 623.5 *μ*g/L (IQR 361-798) in those 20 of 29 septic patients who also had shock. The incidence of AKI in sepsis and septic shock was 28.6% and 65%, respectively.

**Figure 5:**
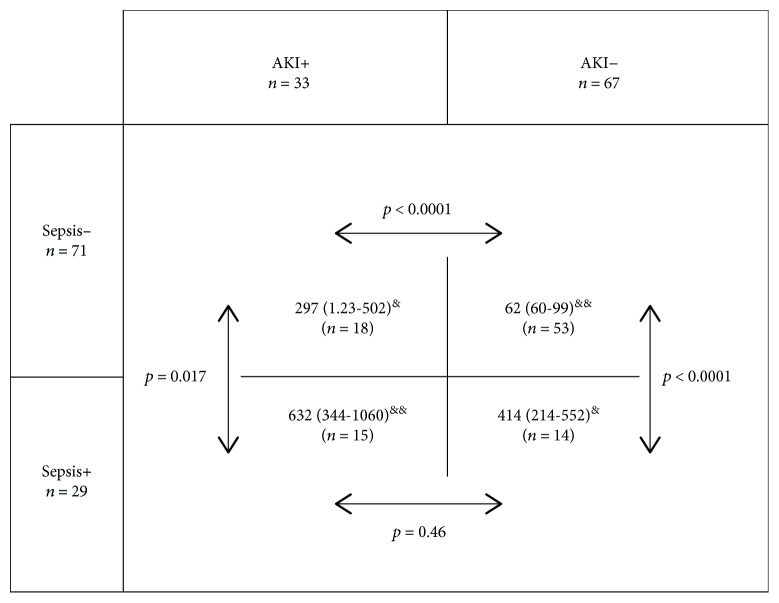
Distribution of admission whole-blood NGAL (*μ*g/L) values at ICU admission depending on AKI and sepsis. AKI+ vs. AKI- and sepsis+ vs. sepsis- represents the presence or absence of either AKI or sepsis upon admission, respectively. Values show median and percentiles 25-75. *p* represents the statistical intragroup differences. wbNGAL values given in *μ*g/L. ^&^Statistical difference between subgroups of nonseptic AKI vs. septic non-AKI patients (*p* = 0.676). ^&&^Statistical difference between subgroups of nonseptic non-AKI vs. septic and AKI patients (*p* < 0.0001). AKI: acute kidney injury.

Serial measurement of wbNGAL did not improve AKI prediction in septic patients compared to nonseptic patients (data not shown). In nonseptic patients, AKI concentrations appeared dependent on AKI status: 62 *μ*g/L (IQR 60-99) in non-AKI and 297 *μ*g/L (IQR 123-502) in AKI patients, *p* < 0.0001. However, when comparing wbNGAL in septic patients with (632 *μ*g/L (IQR 344-1060)) or without (414 *μ*g/L (IQR 214-552)) AKI, there was no statistical difference (*p* = 0.46). Septic patients with and without AKI presented higher wbNGAL than those with the same renal status without sepsis (*p* < 0.0001 for both comparisons). wbNGAL values in septic non-AKI patients were undistinguishable of those of nonseptic AKI patients (*p* = 0.676). Nine of the 11 non-AKI patients with wbNGAL higher than the cut-off >178 *μ*g/L presented sepsis (Figure 5). In our study, there were 20 patients with septic shock: 3 had no AKI (median wbNGAL of 481 *μ*g/L, IQR 142-481 *μ*g/L), 2 had stage 1 (251, IQR 234-269 *μ*g/L), 4 had stage 2 (562, IQR 421-681 *μ*g/L), and 11 had stage 3 (685, IQR 526-1130 *μ*g/L).

## 4. Discussion

The main finding of our study is that admission wbNGAL is a good stratifier of AKI within 48 hours of ICU admission in a heterogeneous group of critically ill patients (AUROC 0.838 (95% CI 0.760-0.917, *p* < 0.0001)). The cut-off value higher than 178 *μ*g/L defines a group of patients with higher severity and higher probability of developing AKI. However, NGAL concentrations were affected by sepsis status irrespective of the AKI presence (*p* = 0.46). Thus, NGAL measures are not useful to evaluate kidney function in patients with sepsis.

Some studies suggested that NGAL in blood or urine could be a useful biomarker of AKI [26]. Most of those initial studies were done in patients after cardiac surgery [7, 27], in context of contrast-induced nephropathy [28], kidney transplantation [18], or chronic kidney disease [29]. Fewer studies were done in general critically ill population, both paediatric [30, 31] and adult patients [19, 21, 32, 33]. There is still debate whether NGAL is a good predictor of AKI in general ICU patients [24]. Our AUROC of wbNGAL is significantly higher than that found by Parikh et al. [18] in the postoperative care in cardiac surgery and by Haase et al. [22] in a paediatric critically ill population. In our study, wbNGAL stratified AKI severity, showing increasing concentrations with an increased KDIGO stage from median values of 71 *μ*g/L in stage 1 and 186 *μ*g/L in stage 2 to 381 *μ*g/L in stage 3 (Figure 2). This increasing pattern in line with the severity of AKI has also been described by other investigators in paediatric and adult patients [24, 25]. Although wbNGAL concentrations in non-AKI patients are similar to those found by Singer et al. [25], AKI severity categories were lower in our study. In view of our results, we would suggest clinicians to consider wbNGAL as a complementary tool in patients at AKI risk or receiving nephrotoxic drugs. Despite NGAL's promising results in general ICU population, the AKIKI [34], ELAIN [35], and the most recent IDEAL-ICU [36] trials showed that wbNGAL combined with KDIGO staging did not improve the timing of renal replacement therapies or patient's prognosis.

In our cohort, the AUROC curve of wbNGAL for AKI prediction showed an optimal cut-off value of 178 *μ*g/L. Our cut-off is close to the ones described in other studies, around 150 *μ*g/L [22, 27, 37]. In critically ill patients, the exact time when acute tubular damage occurs is often unknown. The differences in AUROC described in literature could also be explained by their study designs. Unlike our study, those set up in cardiac surgery or radiocontrast administration had an exact time of a potential onset of renal injury.

We found significant differences in admission wbNGAL between the groups of patients with AKI and those without. We also identified a subgroup of patients who presented high levels of wbNGAL with no increase of creatinine. This subgroup could be considered false positives or, in our opinion, a subgroup of patients who may represent subclinical AKI [22]. Patients with stage 1 showed relatively low wbNGAL concentrations within the group of patients with AKI. This finding could be attributable to treated reversible causes of AKI, like hypovolemia or hypotension. Nickolas et al. [38] described low levels of urine NGAL in patients with prerenal azotaemia. Those patients inadequately resuscitated after a prerenal azotaemia had increasing urinary NGAL concentrations. Similar to our study, this is a clinically relevant finding. Timely treatment of patients with subclinical AKI or stage 1 could avoid AKI progression to failure and tubular damage.

NGAL is a protein upregulated after a tubular injury [39]. However, it can also be produced by other organs like the liver or lung [7] or in different inflammatory situations [40]. In our study, the serial measurement of wbNGAL did not improve AKI prediction in septic patients compared to nonseptic patients. No statistical differences were found between ROC curves obtained at admission and 48 h later; both ROC curves produced very similar cut-offs (Supplemental Table 1). These results concur with those from Bagshaw et al. [41], which showed that peak plasma NGAL did not perform better to predict AKI in septic than in nonseptic patients. This is clinically relevant because single sampling at admission decreases costs, and it is easier to implement in daily routine.

wbNGAL values were higher in patients with sepsis and much higher in those with septic shock. These data strongly suggest that wbNGAL not only is a good predictor of AKI but also can be considered a good severity score in patients with inflammatory status. Although Mishra et al. [27] showed that plasma NGAL was independent of inflammatory markers like C-reactive protein, Zappitelli et al. [31] reported that plasma and urine NGAL concentrations at ICU admission were higher in patients in which AKI was due to sepsis than in those in which it was due to nonseptic causes. Like in our study, these authors found that serial NGAL measurement did not add predictive power to NGAL concentrations at admission. Other articles also suggested that the inflammatory status [24] or septic shock could influence wbNGAL concentrations [22], and this could be found regardless of the presence of AKI [42]. Kim et al. [43] described that wbNGAL was significantly higher in septic patients with AKI regardless of their levels of procalcitonin. In our subgroup of 20 patients with septic shock, 3 of them had no AKI, 2 stage 1, 4 stage 2, and 11 stage 3, with median wbNGAL of 481 *μ*g/L, 251 *μ*g/L, 562 *μ*g/L, and 685 *μ*g/L, respectively. These data favour the hypothesis that inflammatory status could increase the level of wbNGAL and could be misleadingly interpreted as a tubular damage when it has not yet occurred. In these cases, urinary NGAL, which is not submitted to the influence of inflammatory mediators, could have better performance in AKI prediction. Besides, differentiation of monomeric and dimeric plasma NGAL isoforms could be crucial to recognise the NGAL concentration secondary to renal damage or inflammation [44]. Our data also suggest that NGAL may be a biomarker of illness severity. In an article by Shapiro et al. [8], NGAL was a good predictor of septic shock in a panel of multiple biomarkers and correlated with survival. Wang et al. also described that high wbNGAL independently predicted mortality and multiple organ dysfunction syndrome in sepsis and septic shock [45].

On the other hand, a study set up in the emergency department by Wang et al. [46] suggested that combined NGAL and TIMP-1 (tissue inhibitor of matrix metalloproteinases-1, a cell cycle arrest biomarker for AKI) was useful for the diagnosis and risk stratification of patients with AKI, including those who also presented with sepsis. Although cell cycle arrest (CCA) biomarkers are still under evaluation in different clinical settings, their sole predictive power and risk stratification appears to be higher than that exhibited by NGAL. Since CCA biomarkers were presented in Sapphire study [47], there has been an increasing number of studies in paediatric [48] and adult critical care populations, with clinical implementation in rapid response teams [49] and KDIGO care bundles [50, 51]. Besides, unlike NGAL, CCA biomarkers are not determined by sepsis [52, 53].

Our study has some limitations. First of all, the sample size was small, and the study was performed in a single centre. Secondly, we recruited a cohort that might not represent an average ICU population. We aimed to study the AKI incidence in a population with ideally normal baseline renal function. AKI is a common complication of many nonrenal hospitalisations [54–56]. We purposefully excluded patients with a background of chronic disease and/or admitted in the hospital for more than 24 hours prior to ICU admission. However, this is also the main strength of our study, because our unique cohort of patients were not under the effect of intrahospital risk factors for AKI that could have acted as confounding variables. Finally, the point-of-care test used to analyse wbNGAL was not able to differentiate between NGAL isoforms, which could have helped to explain the role of inflammation in wbNGAL concentrations.

## 5. Conclusions

Our study showed that whole-blood NGAL concentrations at ICU admission stratified AKI in adult critically ill patients. Nonetheless, wbNGAL concentrations increased by sepsis status irrespective of AKI occurrence. Thus, NGAL measures should be avoided to evaluate kidney function in patients with sepsis.

## Figures and Tables

**Table 1:** Clinical characteristics of the study patients depending on the cut-off obtained for AKI prediction.

	<178 *μ*g/L	≥178 *μ*g/L	*p* value
*n*	55	45	—
Age (years)	54.9 ± 18.5	64 ± 15.5	0.009
Male	37 (67)	23 (51)	0.075

*Characteristics at ICU admission*			
SAPS II	37.8 ± 14.3	43.15 ± 15.5	0.095
APACHE II	15 ± 6.9	18 ± 8.2	0.059
SOFA	5.2 ± 2.7	7.7 ± 3.6	<0.0001
AKI admission (%)	6 (10.9)	27 (15.6)	<0.0001
KDIGO stage 3 at admission (%)	2 (3.6)	12 (26.7)	0.001
RRT admission (%)	2 (3.6)	3 (6.7)	0.400
Creatinine clearance (mL/min)	109 ± 58	57 ± 52	<0.0001
Cockcroft–Gault	104 ± 47	58 ± 38	<0.0001

*Characteristics during ICU stay*			
Mechanical ventilation (%)	46 (82)	36 (80)	0.410
Vasopressor requirement (%)	6 (11)	22 (49)	<0.0001
ICU length of stay (days)	11.1 ± 9.3	9.4 ± 10	0.360
ICU mortality (%)	10 (18.2)	12 (26.7)	0.210
Sepsis	3 (5.5)	26 (57.8)	0.007
Septic shock	2 (3.6)	18 (40)	<0.0001
AKI development (%)	2 (3.6)	6 (13.3)	0.070
KDIGO stage 3 development (%)	2 (3.6)	16 (35.6)	<0.0001
RRT after admission (%)	0 (0)	4 (8.9)	0.038
RRT total (%)	2 (3.6)	7 (15.6)	0.040

Values expressed as either % per column or mean ± standard deviation. *p*: value of statistical significance. SAPS II: Simplified Acute Physiology Score II; APACHE II: Acute Physiology and Chronic Health Evaluation II; SOFA: Sequential Organ Failure Assessment score; AKI: acute kidney injury; KDIGO: Improving Global Outcomes AKI group classification; RRT: Renal replacement therapies; ICU: intensive care unit.

## Data Availability

The data used to support the findings of this study are restricted by the Hospital de la Santa Creu i Sant Pau's Ethic Committee Board in order to protect patient privacy. Data are available from authors (Dr. Antoni Jordi Betbese Roig, ajbetbese@santpau.cat) for researchers who meet the criteria for access to confidential data.

## Supplementary Materials

Supplementary Materials Supplemental Table 1: diagnostic and overall accuracy of wbNGAL for AKI prediction, RRT at admission, and sepsis at admission.
