# Supplementary material for: Does Whole-Blood Neutrophil Gelatinase-Associated Lipocalin Stratify Acute Kidney Injury in Critically Ill Patients?
Source: Dis Markers. 2019 May 2;2019:8480925. doi: 10.1155/2019/8480925 (PMC6525902; doi:10.1155/2019/8480925)
Supplement: Supplementary Materials — Supplemental Table 1: diagnostic and overall accuracy of wbNGAL for AKI prediction, RRT at admission, and sepsis at admission. [file 8480925.f1.pdf]

Supplemental Table 1. Diagnostic and overall accuracy of wbNGAL for AKI prediction, RRT at admission, and severe sepsis or septic shock at admission *p*: value of statistical significance

Abbreviations: AUC area under curve of ROC analysis; CI confidence interval; AKI acute kidney injury; RRT renal replacement therapies

	AUC (95% CI)	Best Cut-off (ug/L)	Sensitivity (%)	Specificity (%)	*p*
**AKI at admission (n=33)**	0.859 (0.787-0.931)	178	82	73	<0.0001
**AKI development (n=43)**	0.838 (0.760-0.917)	178	77	79	<0.0001
**AKIN 3 at admission (n=14)**	0.833 (0.730-0.936)	240	79	70	<0.0001
**AKIN 3 development (n=18)**	0.861 (0.778-0.945)	264	78	75	<0.0001
**RRT at admission (n=9)**	0.720 (0.588-0.853)	240	67	66	0.030
**Severe sepsis or septic shock at admission (n=29)**	0.870 (0.794-0.947)	195	86	78	<0.0001
